# Impact of inflammation-mediated myocardial fibrosis on the risk of recurrence after successful ablation of atrial fibrillation – the FIBRO-RISK study: Protocol for a non-randomized clinical trial

**DOI:** 10.1097/MD.0000000000014504

**Published:** 2019-03-01

**Authors:** Szilamer Korodi, Rodica Toganel, Theodora Benedek, Roxana Hodas, Monica Chitu, Mihaela Ratiu, Istvan Kovacs, Andras Mester, Imre Benedek

**Affiliations:** a University of Medicine, Pharmacy, Sciences and Technology; b Department of Advanced Research in Multimodality Cardiovascular Imaging, Cardio Med Medical Center; c Emergency Clinical County Hospital, Tirgu Mures, Romania.

**Keywords:** atrial fibrillation, inflammation-mediated myocardial fibrosis, LGE-CMR

## Abstract

**Introduction:**

Interventional ablation has been demonstrated to represent an effective therapy in patients with atrial fibrillation (AF), leading to restoration and maintenance of sinus rhythm in the majority of cases. However, recurrence of AF is encountered in 35% to 40% of cases, and the causes for this frequent complication have not been elucidated so far.

**Material and methods:**

Here we present the study protocol of the FIBRO-RISK trial, a prospective, single-center, cohort study which aims to investigate the impact of inflammatory-mediated myocardial fibrosis on the risk of recurrence after successful catheter ablation of atrial fibrillation. The level of systemic inflammation in the pre-ablation and immediate post-ablation period will be assessed on the basis of serum levels of inflammatory biomarkers (hsCRP, matrix metalloproteases, interleukin-6), while the level of cardiac fibrosis will be determined based on cardiac magnetic resonance imaging associated with complex post-processing techniques for mapping myocardial fibrosis at the level of left atrium and left ventricle. At the same time, the amount of epicardial fat will serve as an indirect marker of localized inflammation and will be determined at different levels in the heart (surrounding left atrium, right atrium or the entire heart), while ventricular function will be assessed on the basis of serum levels of NT-proBNP prior to the procedure. All these parameters will be investigated in patients with successful ablation of AF, who will be divided into 2 groups: group 1 – patients who develop AF recurrence at 1-year, and group 2 – patients with no recurrence of AF at 1-year. In all patients, the following biomarkers will be determined: serum levels of inflammatory biomarkers and NT-proBNP at 24 hours and 1-year post procedure, the amount of myocardial fibrosis at the level of left atrium and left ventricle at baseline +/− 7 days, and the amount of epicardial fat surrounding left atrium, right atrium and the entire heart at baseline +/− 7 days.

The primary endpoint of the study will be represented by the rate of AF recurrence at 1-year post ablation, documented by either ECG or Holter monitoring. The secondary endpoints of the study will consist in:

1. the rate of re-hospitalization,
2. the rate of survival without relapse, and
3. the rate of major adverse cardiovascular events (MACE rate, including cardiovascular death or stroke).

In conclusion, FIBRO-RISK will be the first CMR-based study that will investigate the impact of inflammation-mediated myocardial fibrosis and ventricular remodeling on the risk of recurrence after successful ablation of AF, aiming to validate inflammatory biomarkers and myocardial fibrosis as predictors for AF recurrence.

## 1 Introduction

### 1.1 Background and rationale

Atrial fibrillation (AF) has an increasing prevalence, being currently the most frequent type of supraventricular arrhythmia and representing a significant burden for the healthcare system. In most of the cases, myocardial tissue located at the level of the pulmonary veins represents the origin of AF. The pathophysiology of AF is still incompletely understood, several studies suggesting that profibrotic and inflammatory processes can play a crucial role in the development of AF.^[1]^ So far the success rate of AF ablation is not very high, reaching 70% in patients with paroxysmal AF and 50% in cases with persistent AF. Several risk factors for the occurrence and recurrence of AF have been described in the literature, including left atrial (LA) enlargement, left ventricular (LV) dysfunction, the amount of epicardial fat surrounding cardiac structures, or myocardial fibrosis.^[2]^ However, the relationship between inflammatory-mediated myocardial fibrosis and AF recurrence after radiofrequency catheter ablation has not been elucidated so far.^[3]^

Here we present the study protocol of the FIBRO-RISK trial, a prospective, single-center, cohort study which aims to investigate the impact of inflammatory-mediated myocardial fibrosis on the risk of recurrence after successful catheter ablation of AF.

### 1.2 Study objectives

The *primary objective* of the study is to investigate the correlation between the degree of LA and LV fibrosis quantified by cardiac magnetic resonance (CMR), inflammatory biomarkers, and the risk of AF recurrence post catheter ablation.

The *secondary objective* of the study is to investigate the correlation between structural remodeling of the left and right atrium, epicardial fat tissue volume and serum inflammatory biomarkers in patients with AF undergoing successful catheter ablation and their impact on long term outcomes.

## 2 Methods/design

### 2.1 Study design

This is a clinical prospective, non-randomized, cohort, single-center study to investigate the relationship between inflammatory status, inflammation-mediated atrial and ventricular fibrosis and remodeling, and the risk of AF recurrence. The duration of the study is 2 years which include the initial screening and the follow-up period for the recurrence of AF.

### 2.2 Ethics

The study protocol was approved by the Ethics Committee for Scientific Research of the University of Medicine and Pharmacy of Tirgu Mures (certificate of approval: 346/13.12.2017) and the Ethics Committee for Scientific Research of the Cardio Med Medical Center (certificate of approval 28/10.06.2018). All study procedures comply with the *Declaration of Helsinki* of 1975, and all patients will sign an informed consent and will be checked for the inclusion and exclusion criteria prior to enrollment in the study.

### 2.3 Study population

The study will include 100 subjects with AF suitable for catheter ablation. Imaging biomarkers and laboratory analyses such as: high sensitive C Reactive Protein (hsCRP), matrix metalloproteases (MMPs), interleukin-6 (IL6) and N-Terminal Pro–B-Type Natriuretic Peptide (NT pro-BNP) will be determined in the first 24 hours after the procedure. The anatomy of pulmonary veins, atrial fibrosis, atrial volumes and the amount of epicardial fat will be evaluated and quantified with late gadolinium-enhancement cardiac magnetic resonance (LGE-CMR). All these parameters will be investigated in patients with successful ablation of AF, who will be divided into 2 groups: group 1 – patients who develop AF recurrence at 1 year, and group 2 – patients with no recurrence of AF at 1 year. In all patients, the following biomarkers will be determined: serum levels of inflammatory biomarkers and NT-proBNP at 24 hours and 1-year post procedure, the amount of myocardial fibrosis at the level of left atrium and left ventricle at baseline +/− 7 days, and the amount of epicardial fat surrounding left atrium, right atrium and the entire heart at baseline +/− 7 days.

**Figure 1:**
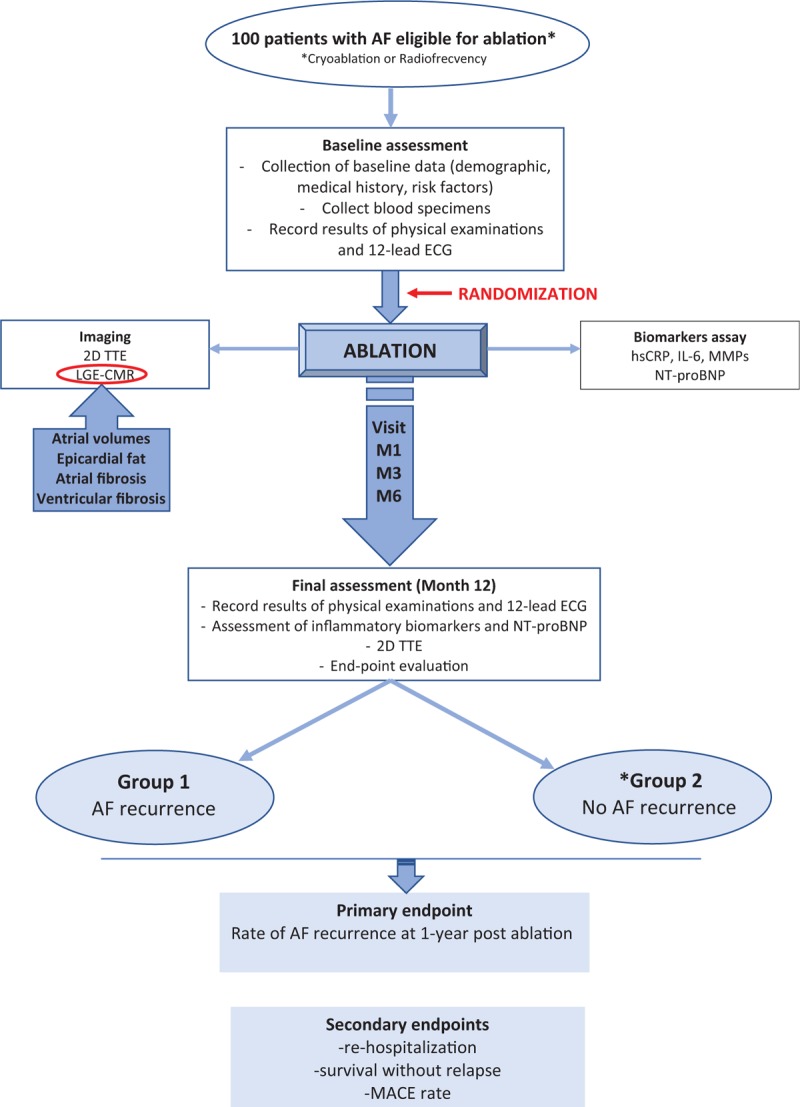
Flowchart diagram of FIBRO-RISK study. hs-CRP = high sensitivity C reactive proteine, IL-6 = Interleukine 6, LGE – CMR = late Gadolinium enhancement CMR, MMPs = matrix metalloproteinases, TTE = transthoracic echocardiography.

The study will be conducted over a period of 2 years, in which patients will be examined at baseline, and will be followed-up for recurrence of AF, as shown by the FIBRO-RISK diagram with the flowchart that will be used in the study (Fig. 1).

Inclusion criteria:

- Patients with non-valvular paroxysmal or persistent AF who undergo successful catheter ablation, either by cryoablation or by radiofrequency advanced 3D mapping system;
- Ability to provide informed consent;
- Patients aged at least 18 years;

Exclusion criteria:

- Patients with valvular AF;
- Patients with acute coronary syndrome in the last 30 days;
- Patients in whom AF is presumed to be caused by hyperthyreosis;
- Patients with long-standing persistent or permanent AF;
- Unwillingness or incapacity to provide informed consent;
- Allergy to gadolinium contrast media;
- Absolute or relative contraindications to magnetic resonance imaging;
- Pregnancy or lactation;
- Women with childbearing potential in absence of any contraceptive treatment;
- Renal insufficiency (creatinine greater than 1.5 mg/dL) or renal failure requiring dialysis;
- Active malignancy or malignancy within the last 5 year prior to enrollment;
- Conditions associated with an estimated life expectancy of under 2 years;

### 2.4 Study settings

The study will be conducted in the Center of Advanced Research in Multimodality Cardiac Imaging of the Cardio Med Medical Center, and is funded by the Romanian Ministry of European Funds, the Romanian Government and the European Union, as part of the research grant number 103545/2016 – CardioIMAGE (contract 43/05.09.2016), which was selected for funding following an international peer-review procedure within the national competition of research grants.

### 2.5 Study groups

One hundred patients with successful ablation of AF who meet the selection criteria will be divided into 2 groups, namely subjects who develop AF recurrence at 1-year post ablation (group 1) and subjects with no recurrence of AF at 1-year post ablation (group 2).

### 2.6 Study procedures and outcome assessment

The following information will be collected from all patients: baseline demographics, history and physical examination findings, cardiovascular risk factors. Conventional 12-lead EGC was performed in each patient at baseline, 1, 3, 6, 12 months follow-up visit.

#### 2.6.1 Biomarkers assays

Complete blood count and biochemistry will be evaluated at the central laboratory of the County Clinical Emergency Hospital of Tirgu Mures as per routine clinical practice, at baseline.

Inflammatory status will be assessed at baseline, respectively at 1-year post procedure. hsCRP levels will be assayed by immunoturbidimetric assay (COBAS INTEGRA 400 plus Analyser, Roche Diagnostics, Switzerland). Matrix metalloprotease 9 and interleukin-6 levels will be assessed via enzyme immunoassay (ELISA) method (Dynex DSX Automated ELISA System, Dynex Technologies, USA; IMMULITE 2000 XPi Immunoassay System, Siemens, USA). Determination of all inflammatory biomarkers will be performed at the Advanced Medical and Pharmaceutical Research Center of the University of Medicine and Pharmacy Targu-Mures. NT-proBNP serum levels will be determined prior to the procedure, at 24 hours, and repeated after 1 year, at the central laboratory of the Emergency Clinical County Hospital of Tirgu-Mures using an electrochemiluminescent immunoassay (IMMULITE 2000 XPi Immunoassay System, Siemens, USA).

#### 2.6.2 2D transthoracic echocardiography

All patients will undergo transthoracic echocardiography with simultaneous ECG recording at baseline and 1-year post ablation procedure. Echocardiographic assessment will be performed with a Vivid E9 echocardiographic equipment (General Electric Vingmed Ultrasound, Horten, Norway), at rest, in the lateral decubitus position. Standard M-mode, 2-dimensional and Doppler images will be acquired as per American Society of Echocardiography standards. Left ventricular volumes and ejection fraction will be quantified and calculated according to Simpson rule, using images acquired in the apical 4-chamber plane. All measurements will be averaged for at least 3 heart cycles.

#### 2.6.3 Late gadolinium-enhancement cardiac magnetic resonance

All CMR examinations will be performed with an 1.5 T Siemens Magnetom Aera magnetic resonance equipment (Siemens, Erlangen, Germany). LV function will be assessed using cine sequences in long-axis (2-chamber and 4-chamber) and short axis. Short-axis volumetry will be used for determination of LV mass, LV ejection fraction, LV end-diastolic volume (LVEDV) and end-systolic volume (LVESV). Ventricular and atrial fibrosis will be assessed using late postgadolinium myocardial enhancement sequences (LGE) at 10 minutes after intravenous injection of gadolinium extracellular contrast agents. Acquired LGE images will be post-processed using the Medis QMass 8.1 software (Medis, Leiden, the Netherlands) with definition of the epicardial and endocardial contours and manual setting of the hyper-enhancement threshold for the recorded sequence of slices. Delayed signal intensity (DSI) analysis performed in 10 to 12 consecutive short-axis LGE images. The full width at half maximum will be used for image segmentation, using an automated hyper-enhancement threshold and fibrotic areas will be considered those with slow washout kinetic.

Quantification of epicardial fat volume will be performed by tracing and manually adjusting epicardial contours, as part of the post-processing protocols.

#### 2.6.4 Ablation protocol

Seldinger technique will be used for venous access, with 1 sheath positioned on the left and 2 sheaths on the right femoral veins. A decapolar diagnostic catheter will be placed in the coronary sinus, after being advanced via left femoral access site and secured with a 6F sheath.

Transesophageal echocardiography will be used to guide interatrial septal puncture. A bolus of 5000 to 7500 U of heparin followed by 1000 to 2000 U/h will be administrated and the following heparin dosage will be adjusted using activated clotting time, determined every 15 to 30 minutes.

In case of radiofrequency ablation, 1 non-steerable sheath will be used for placing the duodecapolar mapping catheter (Lasso) and the other steerable sheath for placing the 3.5 mm tip externally irrigated contact force-sensing ablation catheter. A continuous heparinized saline wash of the long sheaths will be provided during the entire procedure. The 3-D electro-anatomical map will be acquired with the EnSite^TM^ NavX^TM^ system, Model EE3000 (St. Jude Medical, St. Paul, MN, USA), improved by fusing images acquired with CMR images. After inserting the mapping catheter in the ostium of the pulmonary vein (PV), radiofrequency catheter ablation will be performed to obtain pulmonary vein isolation. Ablation will consist in applying a 30 W energy and a 10 to 30 g pressure with temperature adjusted at 50°C, by an irrigated (5–20 ml/min) contact force-sensing catheter positioned on the left atrial wall. A PV will be considered electrically isolated by eliminating all electrical potentials, or by demonstrating successful PV potential dissociation.

In case of cryoablation procedure, a cryoablation balloon (Arctic Front Advance Cryoablation Catheter, Medtronic, Quebec, Canada) will be advanced with transesophageal control via transseptal route and will be placed at the ostium of each pulmonary vein, delivering minus 40 to 70° cooling energy, during 180 to 240 seconds application. The ablation will be considered efficient when complete electrical block between atrial tissue and pulmonary vein will be achieved.

### 2.7 Study time

The clinical trial will be conducted from January 2019 to December 2020.

### 2.8 Outcomes

The *primary outcome* of the study is represented by the rate of AF recurrence at 1-year post ablation, documented by either ECG or Holter monitoring.

*Secondary outcome* refers to rate of re-hospitalization, rate of survival without relapse and rate of major adverse cardiovascular events (MACE rate, including cardiovascular death or stroke)

### 2.9 Participation timeline

- **Baseline (day 0)**
  o Obtain and document consent from participant on study consent form.
  o Verify inclusion/exclusion criteria.
  o Obtain demographic information, medical history, medication history, alcohol and tobacco use history.
  o Record results of physical examinations and 12-lead ECG.
  o Collect blood specimens (complete blood count, biochemistry and inflammatory biomarkers).
  o Imaging procedures: transthoracic 2-D echocardiography, late gadolinium-enhancement CMR
  o Catheter ablation (radiofrequency or cryoablation)
- **Follow-up visits (month 1,3,6)**
  o Record results of physical examinations, 12-lead ECG and medical history.
  o Imaging procedures: transthoracic 2-D echocardiography
- **Final study visit (month 12)**
  o Record results of physical examinations, medical history,12-lead ECG and determination of serum levels of inflammatory biomarkers and NT-proBNP
  o Imaging procedures: transthoracic 2-D echocardiography
  o End-point evaluation.

### 2.10 Study procedures

- Medical history, clinical examination, laboratory tests (complete blood count, biochemistry, serum level of hsCRP, MMP, IL6 and NT-pro-BNP);
- 12-lead ECG
- 2D transthoracic echocardiography with measurement of: cardiac diameters, volumes, valvular function and regurgitation, pressure gradients, left ventricular systolic and diastolic function and ejection fraction.
- Late Gadolinium-Enhancement CMR with the evaluation of: left and right atrial volume, volume of epicardial adipose tissue, degree of atrial and ventricular fibrosis
- Pulmonary vein isolation radiofrequency ablation with advanced 3D mapping system or cryoablation.

### 2.11 Data collection

All the information will be collected in a database that consists of patient's background, medical history, medication, imaging features provided by cardiac ultrasound, CMR and imaging post-processing.

### 2.12 Sample size

The study will include 100 subjects with AF undergoing successful ablation of pulmonary veins via interventional route. After analysis of follow-up data, patients will be distributed in two groups according to the presence or absence of recurrent episodes of AF during the follow-up.

Sample size calculation was performed using the StatMate 2.0 software. For sample calculation, the proportion of event-free population was estimated at 60%. According to this calculation, a sample size of 50 patients in each subgroup has a 90% power to detect an increase in MACE-free rates proportion of 0.29, with a significance level (alpha) of 0.05 (2-tailed). Therefore, the total sample size was established at 100 patients.

### 2.13 Statistical analysis

Statistical analysis will be performed using Graph Pad InStat 3.10 software at the level of significance 5%. All data will be checked for normality. Continuous variables with normal distribution will be presented as mean ± standard deviation and will be compared using *t* test. Non-normally distributed variables will be analyzed using the Mann-Whitney test. Categorical variables will be expressed in numbers and percentages and will be compared using the Fisher exact test.

## 3 Discussion

This study presets the protocol of a prospective, single-center, cohort study which aims to investigate the impact of inflammatory-mediated myocardial fibrosis on the risk of recurrence after successful catheter ablation of AF.

Although AF ablation procedures are constantly improving, recurrences continue to be problematic as a significant proportion of patients present with AF relapse despite an optimum management in the post-ablation period.^[1,4]^ In the latest years, a large number of studies tried to identify predictors for a successful outcome on long term after AF ablation, however reliable clinical markers to predict sinus rhythm maintenance or AF recurrence have not been identified so far. The main contribution of the present study will be to validate new clinical features, such as inflammatory biomarkers, epicardial fat, ventricular remodeling or myocardial fibrosis, as predictors of AF recurrence in patients who are scheduled for catheter ablation. In this trial we hypothesize that several imaging markers associated with cardiac structural alteration, provided by CMR (such as LA, LV fibrosis, atrial volumes or the amount of epicardial fat), associated with serum inflammatory biomarkers (IL-6, MMP-9, hsCRP) could form a powerful prediction panel to predict the risk of AF recurrence in the post ablation period, reflecting the impact of inflammatory-mediated myocardial fibrosis on the risk of arrhythmia.

The relationship between inflammation, myocardial injury and atrial fibrillation has been extensively studied in the recent years.^[5]^ It is well known that a higher amount of atrial fibrotic tissue can favor atrial arrhythmogenesis.^[2,6]^ At the same time, the amount of contrast enhancement in the LA wall, considered to represent the volume of fibrotic tissue of the total LA wall, has been recently described by prospective trials to be independently associated with the likelihood of recurrent arrhythmia after ablation.^[2,6,7]^ Previous studies showed that pre-existing fibrosis on LGE-CMR was the strongest independent predictor of ablation procedure failure and arrhythmia recurrence.^[8]^ McGann et al proved that subjects with >30% atrial fibrosis on LGE images at baseline had a 71% chance of AF recurrence at 1 year.^[9]^

Beside baseline evaluation, several studies suggest that quantification of post ablation LA scarring may play a role in determination of the AF recurrence risk.^[10]^ Several recent studies managed to assess the feasibility of LGE-MRI in detecting both pre-existing and post-ablation atrial and ventricular fibrosis.^[8,11–14]^ Their results raised interest in the use of CMR as a periprocedural risk assessment tool. Despite these promising results, the assessment of atrial and ventricular fibrosis has not yet been widely adopted in clinical practice.^[15–18]^ We should not forget that the capacity of LGE-CMR to detect fibrosis in the atria is still controversial due to the lack of reproducibility. Thus, this trial aims to validate myocardial fibrosis, targeting both atrial and ventricular fibrosis quantified by CMR, as predictor for AF recurrence.

Previous observational studies showed that elevation of several inflammatory serum biomarkers, such as hsCRP or IL-6, are associated with occurrence and recurrence of AF, since patients with AF present significantly higher levels of inflammatory biomarkers as compared with subjects maintaining sinus rhythm.^[3,19–22]^ Therefore, the role of inflammation in AF recurrence has been recently proposed as a new and challenging hypothesis to explain the high rate of recurrence after AF conversion.^[3,21,23–26]^ As the exact pathways from inflammation to AF are still unclear, it is presumed that inflammatory cytokines may bind to atrial myocytes inducing tissue damage.^[24]^ In line with this hypothesis, the FIBRO-RISK trial aims to investigate the correlation between inflammatory biomarkers and cardiac fibrosis, evaluated via LGE-CMR, in order to prove the impact of inflammatory-related myocardial fibrosis on the risk of recurrence after successful catheter ablation of AF.

Besides inflammation, neurohormonal activation, classically associated with ventricular dysfunction, has been shown to play a significant role in the recurrence of AF following pulmonary vein isolation.^[20,21,23–26]^ A very recent study demonstrated that in patients admitted in an intensive care facility, the presence of ventricular remodeling leading to ventricular dysfunction was associated with a higher incidence of AF and conduction abnormalities.^[27]^ NT-proBNP is a biomarker reflecting ventricular dysfunction, which has been shown to predict AF recurrence unrelated to ablation procedure.^[28,29]^ However, it still remains unclear if these biomarkers are useful predictors of AF recurrence after ablation. In the FIBRO-RISK study, we aim to validate, for the first time in the literature, the role of ventricular remodeling, serum biomarkers associated with ventricular dysfunction, inflammation and myocardial fibrosis, assessed by LGE-CMR, as independent predictors of AF recurrence after successful catheter ablation, in order to prove that inflammation can play a significant role in the complex interrelation between myocardial dysfunction, remodeling and AF recurrence.

## 4 Conclusions

FIBRO-RISK will be the first CMR-based study that will investigate the impact of inflammation-mediated myocardial fibrosis on the risk of recurrence after successful catheter ablation of AF, aiming to validate inflammatory biomarkers and myocardial fibrosis as predictors for AF recurrence.

## Author contributions

Submission to ethical committee was performed by Korodi Szilamer. The radiologist responsible for CMR imaging interpretation is Mihaela Ratiu. Korodi Szilamer, Roxana Hodas and Theodora Benedek will perform data statistical analysis. All the authors approved the final manuscript.

**Conceptualization:** Szilamer Korodi, Rodica Toganel, Roxana Hodas, Mihaela Ratiu, Andras Mester, Istvan Kovacs, Imre Benedek.

**Data curation:** Theodora Benedek, Kovacs Istvan.

**Formal analysis:** Andras Mester, Theodora Benedek.

**Investigation:** Mihaela Ratiu, Theodora Benedek, Istvan Kovacs.

**Methodology:** Szilamer Korodi, Rodica Toganel, Imre Benedek, Theodora Benedek.

**Resources:** Imre Benedek.

**Supervision:** Imre Benedek, Theodora Benedek.

**Validation:** Mihaela Ratiu.

**Visualization:** Mihaela Ratiu, Andras Mester.

**Writing – original draft:** Szilamer Korodi, Rodica Toganel, Roxana Hodas, Mihaela Ratiu, Andras Mester, Istvan Kovacs, Imre Benedek.

Rodica Toganel orcid: 0000-0001-6510-9646.
